# Spatio-Temporal Context, Correlation Filter and Measurement Estimation Collaboration Based Visual Object Tracking

**DOI:** 10.3390/s21082841

**Published:** 2021-04-17

**Authors:** Khizer Mehmood, Abdul Jalil, Ahmad Ali, Baber Khan, Maria Murad, Khalid Mehmood Cheema, Ahmad H. Milyani

**Affiliations:** 1 Department of Electrical Engineering, International Islamic University, Islamabad 44000, Pakistan; abdul.jalil@iiu.edu.pk (A.J.); baber.khan@iiu.edu.pk (B.K.); maria.murad@iiu.edu.pk (M.M.); 2 Department of Software Engineering, Bahria University, Islamabad 44000, Pakistan; ahmad.buic@bahria.edu.pk; 3 School of Electrical Engineering, Southeast University, Nanjing 210096, China; 4 Department of Electrical and Computer Engineering, King Abdulaziz University, Jeddah 21589, Saudi Arabia; ahmilyani@kau.edu.sa

**Keywords:** spatio-temporal context, object tracking, scale correlation filter, extended kalman filter

## Abstract

Despite eminent progress in recent years, various challenges associated with object tracking algorithms such as scale variations, partial or full occlusions, background clutters, illumination variations are still required to be resolved with improved estimation for real-time applications. This paper proposes a robust and fast algorithm for object tracking based on spatio-temporal context (STC). A pyramid representation-based scale correlation filter is incorporated to overcome the STC’s inability on the rapid change of scale of target. It learns appearance induced by variations in the target scale sampled at a different set of scales. During occlusion, most correlation filter trackers start drifting due to the wrong update of samples. To prevent the target model from drift, an occlusion detection and handling mechanism are incorporated. Occlusion is detected from the peak correlation score of the response map. It continuously predicts target location during occlusion and passes it to the STC tracking model. After the successful detection of occlusion, an extended Kalman filter is used for occlusion handling. This decreases the chance of tracking failure as the Kalman filter continuously updates itself and the tracking model. Further improvement to the model is provided by fusion with average peak to correlation energy (APCE) criteria, which automatically update the target model to deal with environmental changes. Extensive calculations on the benchmark datasets indicate the efficacy of the proposed tracking method with state of the art in terms of performance analysis.

## 1. Introduction

Visual object tracking (VOT) has emerged as a dynamic study area due to its utilization in a wide range of applications such as human action recognition [1,2,3], traffic monitoring [4,5], pellet ore phase [6], smart city [7], embedded system [8], surveillance [9,10,11] and medical diagnosis [12,13]. While significant progress has been made in recent years, accurate estimation for tracking an object is still a challenge in a video sequence due to various factors such as scale variations, occlusion, deformation, background clutters, to name a few [14,15,16]. Target tracking methods, being classified as generative [17] and discriminative [18], are widely referred in literature with prominent applications. Generative tracking methods learn the appearance model of the target and search for the highest matching score. These methods achieve good tracking results at the expense of computational cost. Discriminative tracking methods treat it as binary classification and achieve favorable results. However, tracking in these methods might get affected when training data is small.

### 1.1. Related Work

Rich literature is available in visual object tracking dealing with target appearance models and model updating. In this section, three types of tracking algorithms mainly related to our tracking method are introduced: tracking by spatio-temporal context (STC), tracking by correlation filter, and tracking by Kalman filter (KF).

#### 1.1.1. Tracking by STC

A tracking algorithm based on STC utilizes fast Fourier transform (FFT) to accelerate the calculations. Zhang et al. [19] proposed a spatio-temporal context (STC) based tracking model by formulating temporal relation between the target and its context in a Bayesian framework. Afterward, the model’s confidence map is maximized to determine the target location by further updating the tracking model and scale. Based on [19], Zhang et al. proposed an adaptive STC model for online tracking by incorporating a histogram of oriented gradients (HoG) features and color naming (CN) features in the STC framework. They also used the average difference between adjacent frames to adjust the learning rate when the model is updated [20]. To further improve tracking performance in the STC framework, Wang et al. [21] proposed an improved tracking model that combines STC with a convolutional neural network (CNN) to extract online CNN’s deep characteristics without training. A motion vector-based mechanism for predicting target position under motion is incorporated in the STC framework to improve the STC scale. It also combined a scale correlation filter with STC to extract different scale samples around the target and used the HoG operator to form a pyramid of scale characteristics [22,23].

#### 1.1.2. Tracking by Correlation Filter

Correlation filters have been broadly applied in object tracking [24,25,26,27,28]. To solve scale estimation in correlation filtering, Danelljan et al. [29] proposed a tracker based on correlation filters for translation and scale in image scale pyramid representation. Implementation in [29] is optimized by using various strategies for reducing computational cost in [30]. Zhang et al. [31] used [30] as its base tracker and proposed motion aware correlation filters (MACF) based tracking method by incorporating joint motion estimation based Kalman filter in discriminative correlation filters and used confidence of squared response map (CSRM) criteria for model update and occlusion detection. Ma et al. [32] used implementation in [30] and proposed a fast and accurate scale estimation method by incorporating average peak to correlation energy (APCE) in a multiresolution translation filter. Li et al. [33] included a scale adaptive tracking method in KCF framework. They address the issue of fixed template size in KCF and incorporated HoG and CN features.

#### 1.1.3. Tracking by Kalman Filter

Kalman filters are widely utilized for occlusion handling in various trackers [34,35,36,37,38]. Yang et al. [39] proposed an improved STC algorithm and combined the Kalman filter with STC making it more robust and used Euclidean distance to detect occlusion. Mehmood et al. [40] proposed a tracking algorithm similar to [39]. In their implementation, they have incorporated context-aware formulation and combined Kalman filter in the STC framework. Moreover, they used the maximum value of the response map for occlusion detection. Khan et al. [41] proposed an improved tracker based on long-term correlation tracking (LCT). They incorporated the Kalman filter in the LCT framework for occlusion handling and peak to side ratio (PSR) of response map for occlusion detection.

Based on the presented literature, it can be concluded that significant modifications have been made in the STC algorithm. These modifications are in terms of occlusion detection and handling mechanisms, target model update mechanisms, incorporation of scale update schemes, the fusion of various cues and features, along with deep learning techniques and adaptive learning rate mechanisms. In this article, all the tracking results of the proposed method are available on Google Drive Link:

https://drive.google.com/drive/folders/1nRiUyLfXkBk6tYcSuJaqkW1WAEiyDOX2?usp=sharing.

### 1.2. Our Contributions

Based on related work, this article proposes an object tracking algorithm that enhances STC under scale variations, background clutter, occlusion, illumination variation, and deformation. The contributions of this article are numerous, described as follows:

(1) We propose a scale correlation filter-based pyramid representation mechanism to accurately extract the target without accumulating the scale model’s error. We use a combination of spatio-temporal context and scale correlation filter to achieve accurate object tracking.
(2) We introduce an effective method in which the object can be tracked accurately by utilizing extended Kalman filter (EKF) detection for nonlinear target motion. We also use the response map’s peak value to measure the reliability of the current estimated position. If the tracking result is unreliable, this method can regain the target position to continue tracking.
(3) We propose an adaptive learning rate mechanism based on the average peak to correlation energy (APCE) based on the target appearance model. This method can effectively prevent the tracking model from the wrong appearance.
(4) Experimental results have been presented on de facto standard videos to show the efficacy of the proposed method over STC [19], DCF_CA_ [26], Modified KCF [28], MACF [31], Modified STC [40], and AFAM-PEC [41].

A correlation filter-based discriminative scale mechanism is incorporated in spatio-temporal learning in the proposed work, making it robust and effective in scenarios such as clutter background, illumination variation, scale variations, and fast motion. The adaptive learning rate mechanism is based on APCE between consecutive frames. It is fused in this framework so that the tracking model can be updated according to the target’s shape and motion. If the model is updated on a fixed learning rate, it does not cope with the target’s shape, thereby losing it in the subsequent frames.

The extended Kalman filter aspect, which is utilized in the current study, is when the target undergoes occlusion. The condition to decide whether the target is occluded is based on the response map’s maximum value. In the proposed tracker, not only extended Kalman filter is applied, but a mechanism is also devised for its activation in the STC framework, making it better both qualitatively and quantitatively than various trackers.

The current study focuses on addressing limitations in the spatio-temporal context framework by incorporating efficient scale-space formulation, occlusion detection, and handling and adaptive learning rate modules.

### 1.3. Paper Outline

This paper’s organization is as follows: a brief explanation of spatio-temporal context, tracking is given in Section 2. Section 3 defines scale correlation filter, extended Kalman filter, occlusion detection method, and adaptive learning rate mechanism by explaining the proposed method for online tracking. Experiment parameters are discussed in Section 4. Performance analysis is discussed in Section 5. Section 6 includes a discussion, while Section 7 concludes the paper.

## 2. Spatio-Temporal Context Tracking

**Figure 1:**
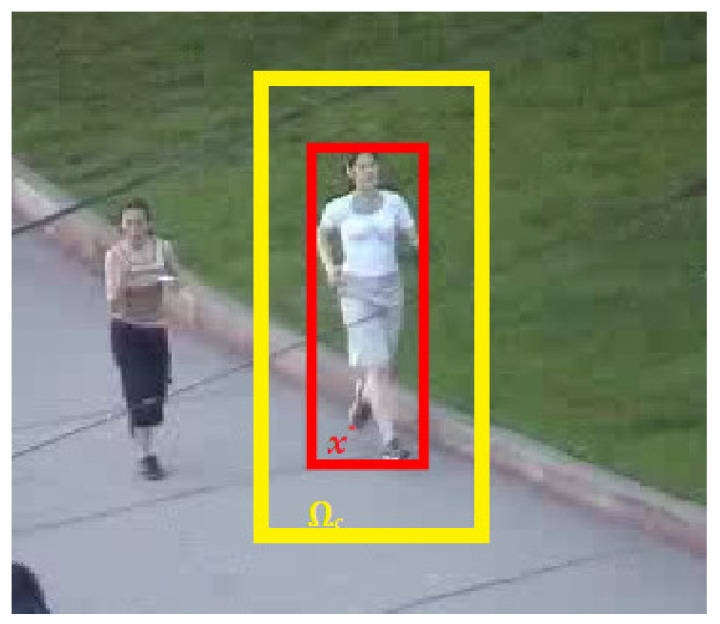
Spatial relation between an object and its context.

STC tracking algorithm is based on the Bayesian framework for finding target location by utilizing context information. In every frame, the confidence map is maximized to compute the target center. The feature set around the target location in each frame is defined as $X^{c}=\{n\left(o\right)=\left(I\left(o\right),o\right)|o\;\in \;$Ω_c_ (x*)} where I(o) is the image greyscale value at location o while Ω_c_ (x*) is the context around target center x*. It is shown in Figure 1.

To formulate the tracking problem, the confidence map is computed for estimation of the likelihood of target location:

(1) $$n\left(x\right)=P\left(x|j\right)=\sum_{n\left(o\right)\in X^{c}}\;P\left(x,n\left(o\right)|j\right)=\sum_{n\left(o\right)\in X^{c}}\;P\left(x,n\left(o\right)|j\right)P\left(n\left(o\right)|j\right)$$

where x is the target coordinates and j is the target, P(n(o)|j) is the context prior model that represents the features of context appearance. P(x,n(o)|j) is the spatial context model that formulates spatial relation between target position and its context. It identifies and resolves uncertainties for different image measurements. Confidence map function n(x) is defined as follows:

(2) $$n\left(x\right)=P\left(x|j\right)={\;m\;e}^{\left(-\left|\frac{x-x^{*}}{\theta }\right|\xi \right)}$$

where m is constant for normalization, ξ is shape parameter, and θ is scale parameter. Appropriate selection of shape parameters helps the spatial context model learn. Setting ξ > 1 results in oversmoothing of confidence map near the center. If ξ < 1 sharp peak response is generated while learning spatial context. Due to these issues, STC uses ξ = 1. Context prior model needs to be calculated before learning spatial context model. Spatial context is modeled by the image intensity function and Gaussian weighted function mentioned in (3) and (4).

(3) $$P\left(n\left(o\right)|j\right)=I\left(o\right)\omega_{\gamma }\left(o-x^{*}\right)$$

(4) $$\omega_{\gamma }={c\;e}^{\left(-\frac{{\left|x-x^{*}\right|}^{2}}{\sigma^{2}}\right)}$$

where σ is scale representation. (4) is restricted between 0 and 1 by using its normalization constant c. The closer the context location j is to the current target location $x^{*}$, the larger the weight should be set to predict the target location in the next frame. (5) defines the spatial context model.

(5) $$P\left(x,n\left(o\right)|j\right)=h^{\mathrm{sc}}\left(x-o\right)$$

Solving for spatial context.

(6) $$=h^{\mathrm{sc}}\left(x-o\right)I\left(o\right)\omega_{\gamma }\left(o-x^{*}\right)$$

(7) $$=h^{\mathrm{sc}}\left(x\right)⊗\left(I\left(x\right)\omega_{\gamma }\left(x-x^{*}\right)\right)$$

where ⊗ denotes convolution operation. Fast Fourier transform (FFT) is used for improving speed and it can be calculated as follows:

(8) $$ℱ\left(n\left(x\right)\right)=ℱ\left(h^{\mathrm{sc}}\left(x\right)\right)⊙F\left(I\left(x\right)\omega_{\gamma }\left(x-x^{*}\right)\right)$$

where ⊙ denotes element-wise multiplication. Solving (8) for the spatial context model.

(9) $$h^{\mathrm{sc}}\left(x\right)={ℱ}^{-1}\left(\frac{F\left({\mathrm{me}}^{-\left|\frac{x-x^{*}}{\theta }\right|\xi }\right)}{F\left(\left(I\left(x\right)\omega_{\gamma }\left(x-x^{*}\right)\right)\right)}\right)$$

where ${ℱ}^{-1}$ denotes inverse FFT in (9). In the STC model, the target is initialized position at the first frame. The spatial context model $h^{\mathrm{sc}}$ learns relatively spatial relations between different pixels in the Bayesian framework. For subsequent frames, the STC model $H_{t+1}^{\mathrm{stc}}\left(x\right)$ can be updated by using the spatial context model $h_{t}^{\mathrm{sc}}\left(x\right)$. By computing the extreme of the confidence map center position of the target $x_{t+1}^{*}$ at (t + 1) the frame can be attained as given in (10).

(10) $$x_{t+1}^{*}=\arg_{x\in {Ω}_{c}\left(x_{t}^{*}\right)}{\;\max\;n}_{t+1}\left(x\right)$$

Similarly, a confidence map can be calculated from (11).

(11) $$n_{t+1}\left(x\right)={ℱ}^{-1}\left(F\left(H_{t+1}^{\mathrm{stc}}\left(x\right)\right)⊙ℱ\left(I_{t+1}\left(x\right)\omega_{\gamma }\left(x-x_{t}^{*}\right)\right)\right)$$

STC model is updated on learning rate ρ as mentioned in (12).

(12) $$H_{t+1}^{\mathrm{stc}}=\left(1-\rho \right)H_{t+1}^{\mathrm{stc}}+\rho h_{t}^{\mathrm{sc}}$$

where ρ is the learning rate and $h_{t}^{\mathrm{sc}}$ is the spatial context model computed in (9).

## 3. Proposed Tracker

**Figure 2:**
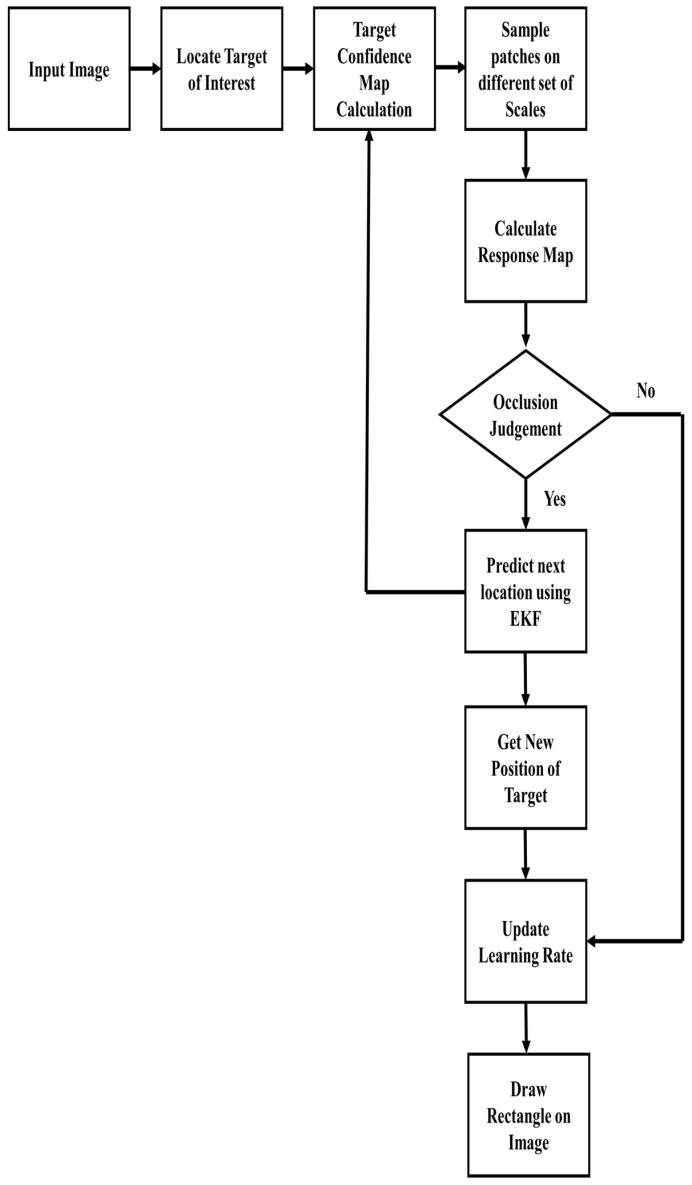
Flowchart of the proposed tracking model.

In this section, the proposed tracker will be discussed. First, a correlation filter-based adaptive scale scheme is discussed. Second, an extended Kalman filter-based occlusion handling mechanism is investigated. Third, an adaptive learning rate scheme is presented. The execution scheme of the proposed tracker is shown in Figure 2. In each image sequence, the target of interest’s location is initialized manually on the first frame from the given ground truth. Afterward, the target confidence map is calculated. Sample patches of a different set of scales are estimated from the confidence map of STC. Then, the maximum value of the response map is calculated. If the response map’s value is less than the fixed threshold, then the extended Kalman filter is activated. The Kalman filter will predict the location of next frame and update the tracking model during this entire period. Once the response map’s value exceeds the fixed threshold, then the Kalman filter is deactivated. Afterward, the learning rate is updated, and the target entire tracking model is updated based on the calculated position.

Different variables and notations used in the following sections are presented in Table 1.

### 3.1. Scale Space Tracking

Discriminative correlation filters are widely used in visual object tracking. For estimating the target scale, a scale correlation filter-based tracking model is used. It first extracts different scale samples around the target position; then, the HOG feature pyramid sample is extracted from the location. For finding an optimal correlation filter, cost function given in (13) needs to be minimized.

(13) $$\varepsilon ={\left\|\sum_{l=1}^{d}h^{l}*f^{l}-g\right\|}^{2}+\lambda \sum_{l=1}^{d}{\left\|h^{l}\right\|}^{2}$$

where g is desired output, λ is the regularization term, ∗ is circular convolution operator, h is the HOG features after extracting from the sample, l indicates l-dimensional HOG features, g indicates two-dimensional Gaussian function, d indicates the total dimension of HOG features, and f is the correlation filter. The solution of (13) in the frequency domain is given in (14).

(14) $$H^{l}=\frac{{\bar{G}F}^{l}}{\sum_{k=1}^{d}\bar{F^{k}}F^{k}+\lambda }$$

By minimizing output error over training patches, an optimal filter can be obtained. However, it is not suitable for online tracking because of computational cost. For efficient tracking, the numerator and denominator of the correlation filter $H^{l}$ are updated separately as given in (15) and (16).

(15) $$A_{t}^{l}=\left(1-\gamma \right)A_{t-1}^{l}+\gamma \bar{G_{t}}F_{t}^{l}$$

(16) $$B_{t}=\left(1-\gamma \right)B_{t-1}+\;\gamma \sum_{k=1}^{d}\bar{F_{t}^{k}}F_{t}^{k}$$

where γ is the learning rate. By maximizing the correlation score, the target state can be determined as given in (17).

(17) $$y=F^{-1}\left\{\frac{\sum_{l=1}^{d}\bar{A^{l}}Z^{l}}{B+\lambda }\right\}$$

where $Z^{l}$ denotes HOG features extracted from prediction

### 3.2. Extended Kalman Filter

Within the visual object tracking research area, EKF is widely used to estimate the system. The target location problem can be viewed as an estimation problem, as it provides measurement-based prediction. For the current estimate, EKF linearizes the nonlinear equations. Afterward, EKF is applied to that linearized model [42]. EKF involves two steps which are prediction and correction. During prediction, state and covariance estimates are computed for the current frame using (18) and (19).

(18) $$x_{t}^{-}={A\hat{x}}_{t-1}+{\mathrm{Bu}}_{t}$$

(19) $$P_{t}^{-}={\mathrm{AP}}_{t-1}A^{T}+Q$$

where $x_{t}^{-}$ is the state target vector, $Bu_{t}$ is noise, A is process Jacobian, Q is process noise covariance, and $P_{t}^{-}$ is the predicted error covariance. During the correction, Kalman gain $K_{t}$ is calculated. It balances between prior estimation uncertainty and measurement noise as given in (20).

(20) $$K_{t}=P_{t}^{-}J_{H}^{T}{\left(J_{H}P_{t}^{-}J_{H}^{T}+R\right)}^{-1}$$

where $J_{H}$ is measurement Jacobian and R is measurement noise. State estimate is updated using prior estimate and error between measurement and predictive measurement as given in (21).

(21) $$x_{t}={\hat{x}}_{t}^{-}+K_{t}\left(z_{t}-J_{H}{\hat{x}}_{t-1}^{-}\right)$$

The difference $\left(z_{t}-J_{H}{\hat{x}}_{t-1}^{-}\right)$ is called measurement innovation or residual. It reflects the discrepancy between predicted measurement $J_{H}{\hat{x}}_{t-1}^{-}$ and actual measurement $z_{t}$.

Posteriori estimation of variance in given in (22).

(22) $$P_{t}=\left(I-K_{t}J_{H}\right)P_{t}^{-}$$

where $P_{t}$ is the updated error covariance, $J_{H}$ is matrix related to the measurement of the state and $K_{t}$ is the updated Kalman gain.

### 3.3. Occlusion Detection

When the target undergoes occlusion, then the STC model is updated incorrectly, thereby losing the target. The maximum value of the target map is used to detect occlusion, which changes its value with the target state’s situation. If the target is occluded, then the value of the response map is small. However, when the target reappears then its value increases. The value of response map determines whether the target is tracked by improved STC or by EKF. For given input image sequence first the confidence map is computed in the frequency domain. If the target is severely occluded, then EKF will predict the position and update improved STC using a feedback loop for the next frame.

### 3.4. Adaptive Learning Rate

The model is updated adaptively by using average peak to correlation energy (APCE) [43]. It is defined in (23).

(23) $${\mathrm{APCE}}_{t}=\frac{{\left|f_{\max}-f_{\min}\right|}^{2}}{\mathrm{mean}\left(\sum_{w,h}{\left(f_{w,h}-f_{\min}\right)}^{2}\right)}$$

where $f_{\max}$ is maximum response value, $f_{\min}$ is minimum response value and $f_{w,h}$ is corresponding row and column value of response map. APCE specified the degree of fluctuation between response maps and detected targets. (24) gives expression of model update.

(24) $$\{\begin{array}{c} {\mathrm{bz}}_{t}=\frac{{\mathrm{APCE}}_{t}}{{\mathrm{APCE}}_{0}}\\ \gamma_{t}=\gamma_{0},\;\;{\;\mathrm{bz}}_{t}>{\mathrm{bz}}_{0}\\ {\;\gamma }_{t}=\gamma_{0}\cdot {\mathrm{bz}}_{t},\;\;\mathrm{otherwise} \end{array}$$

where ${\mathrm{APCE}}_{t}$ is the value at *t*-th frame, ${\mathrm{APCE}}_{0}$ is the value at the initial frame and $bz_{0}$ is threshold to decide learning rate.

Algorithm 1 is presented below.
**Algorithm 1:** Proposed Tracker at time step t**Input**: Image Sequence of n Frames. Position of Target at First Frame. **Output:** Target Position for each frame in Image Sequence. *for* frame 1 to n frames.
(1)Calculate context prior model using (3).(2)Calculate confidence map using (11).(3)Calculate target center.(4)Calculate translation correlation using (17).(5)Calculate the maximum value of response map.(6)**if** response map < threshold(7)new position = Kalman prediction(8)**end**(9)Calculate Kalman gain using (20).(10)Estimate position for next frame using (21).(11)Estimate error covariance using (22).(12)Calculate APCE using (23).(13)Update model using (24).(14)Calculate scale correlation using (17).(15)Update translation and scale model using (15) and (16).(16)Update context prior model using (3).(17)Update spatial context model using (9).(18)Update spatio-temporal context model using (12).(19)Calculate the target position for each frame.(20)Draw a rectangle on the target in each frame.
*End*

## 4. Experiments

To evaluate the performance of the proposed tracker both qualitatively as well as quantitatively, extensive experiments were conducted on image sequences selected from Temple Color (TC)-128 [44], OTB2013 [45], OTB2015 [46], and UAV123 [47] datasets. Challenging factors associated with these sequences are scale variations, deformation, partial or full occlusions, background clutter, illumination variations, and fast motion.

### 4.1. Evaluation Criteria

The proposed tracker was compared quantitatively with existing tracking methods based on distance precision rate (DPR) and center location error (CLE). CLE is defined as Euclidean distance calculated between the tracker and ground truth of target. The calculation formula is mentioned in (25).

(25) $$\mathrm{CLE}=\sqrt{{\left(x_{i}-x_{gt}\right)}^{2}+{\left(y_{i}-y_{gt}\right)}^{2}}$$

where ($x_{i,},y_{i}$) are positions calculated by tracking algorithm and ($x_{gt},y_{gt}$) are ground truth values. DPR is the percentage of frames when distance threshold is greater than the estimated CLE.

### 4.2. Parameter Settings

We set the same parameter values as in [19] and [29]. Map function parameters α and β were set to 2.25 and 1, respectively [19]. Regularization weight parameter λ was set to 0.01. Standard deviation of desired scale filter output was 0.25. Number of scales was 33, and scale factor was 1.02 [29]. We set these values as they turned out to be the best setting in our implementation. However, changing these parameters leads to inferior performance of tracker. The threshold of DPR is 20 pixels.

## 5. Performance Analysis

### 5.1. Quantitative Analysis

DPR comparison is given in Table 2. In sequences (Baby_ce, Car9, Carchasing_ce4, Crossing, Jogging2, Ring_ce, Singer1, Tennis_ce2, and Tennis_ce3), the proposed tracker outperforms Modified KCF, STC, MACF, and DCF_CA_. In sequences (Building3, Carchasing_ce3, Cardark, Cup, Juice, Man, Plate_ce2, and Sunshade), all tracking methods have similar performance. In sequences (Bike3, Busstation_ce2, Car4, Girl2, Guitar_ce2, Human3, Jogging1, Skating2, and Walking2), the proposed has slightly less precision value. However, the proposed has a higher mean value than other tracking methods.

The average center location error comparison is given in Table 3. In sequences (Baby_ce, Car4, Carchasing_ce4, Cardark, Crossing, Plate_ce2, Singer1, Tennis_ce2 and Tennis_ce3) the proposed tracker outperforms Modified KCF, STC, MACF and DCF_CA_. In sequences (Bike3, Building3, Busstation_ce2, Carchasing_ce3, Cup, Girl2, Guitar_ce2, Human3, Jogging1, Jogging2, Juice, Man, Ring_ce, Skating2, Sunshade, and Walking2), the proposed tracker has a slightly high error value. However, the proposed tracker has the lowest mean error compared to the other tracking methods.

The frame per second (FPS) comparison is given in Table 4. In sequences (Baby_ce, Building3, Car4, Carchasing_ce3, Carchasing_ce4, Cardark, Crossing, Cup, Jogging 2, Man, Plate_ce2, Tennis_ce2, and Tennis_ce3), the proposed tracker outperforms Modified KCF, STC, MACF, AFAM-PEC, Modified STC, and DCF_CA_ in terms of accuracy. However, the frame rate of the proposed tracker is low in comparison with other tracking methods.

**Figure 3:**
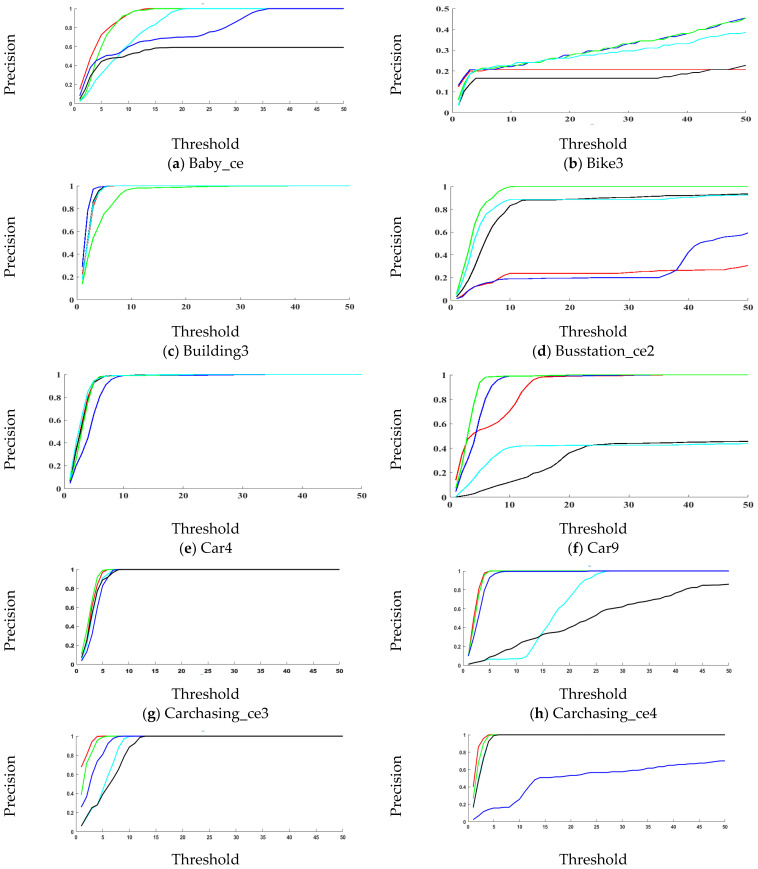

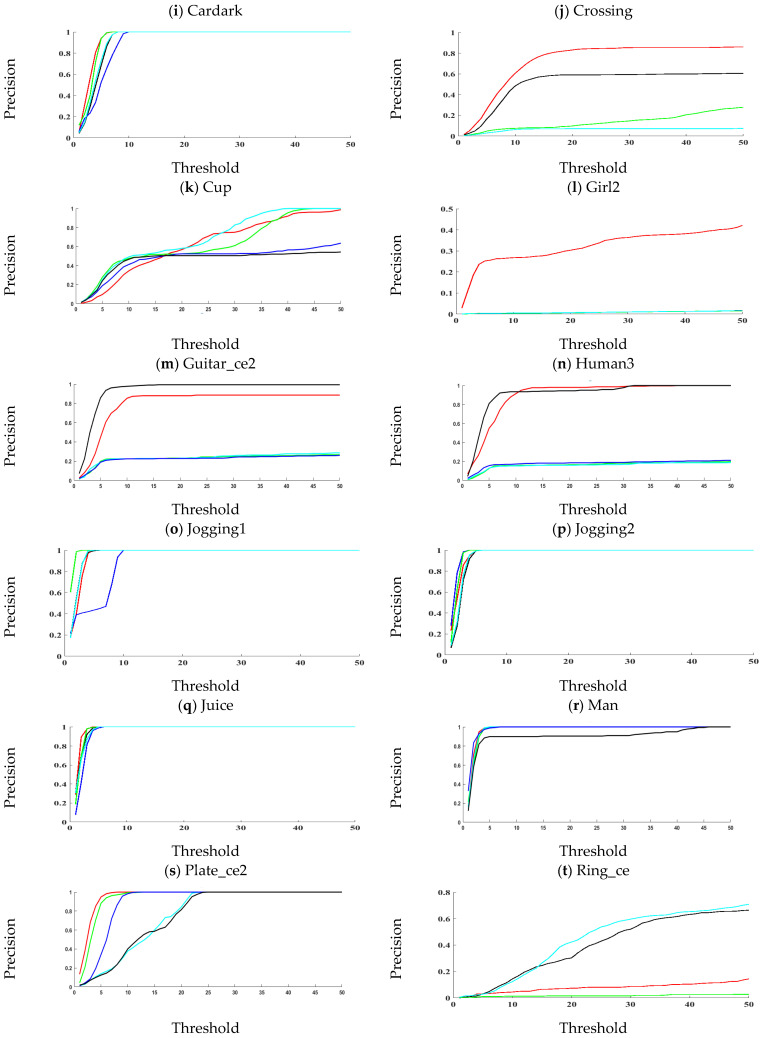

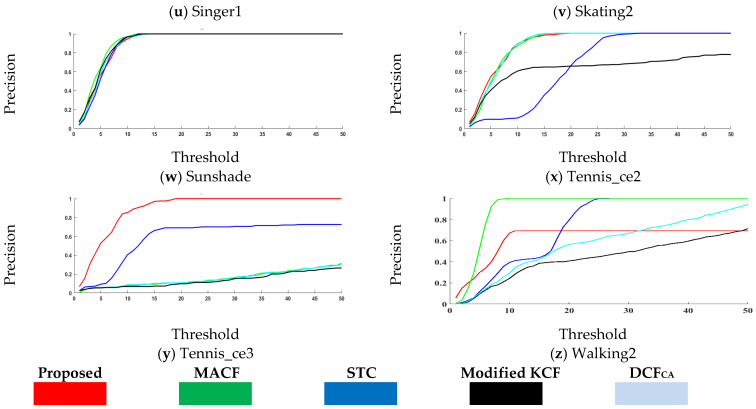
Precision plots comparison on TC-128, OTB2013, OTB2015, and UAV123 datasets.

The precisions plots are shown in Figure 3. Table 2 provides the mean precision value of the tracker in the entire image sequence. However, the tracker might get drift for a few frames and then recover itself. Therefore, to review tracker performance during the whole image sequence, these plots are presented. Various challenges were present in sequences such as occlusion, scale variations, deformation, etc. In sequences (Baby_ce, Carchasing_ce3, Car4, Cardark, Carchasing_ce4, Crossing, Cup, Jogging1, Jogging2, Guitar_ce2, Man, Plate_ce2, Ring_ce, Singer1, Sunshade, Tennis_ce2, and Tennis_ce3), the proposed tracker has the highest precision in the entire sequence. In sequences (Bike3, Building3, Busstation_ce2, Car9, Girl2, Human3, Juice, Skating2, and Walking2), the proposed tracker has slightly low precision.

**Figure 4:**
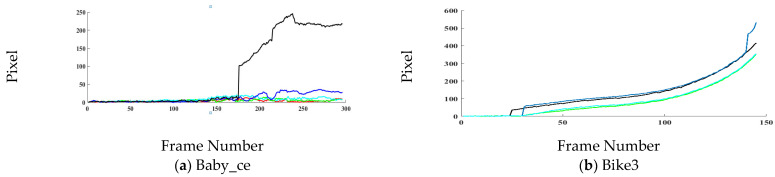

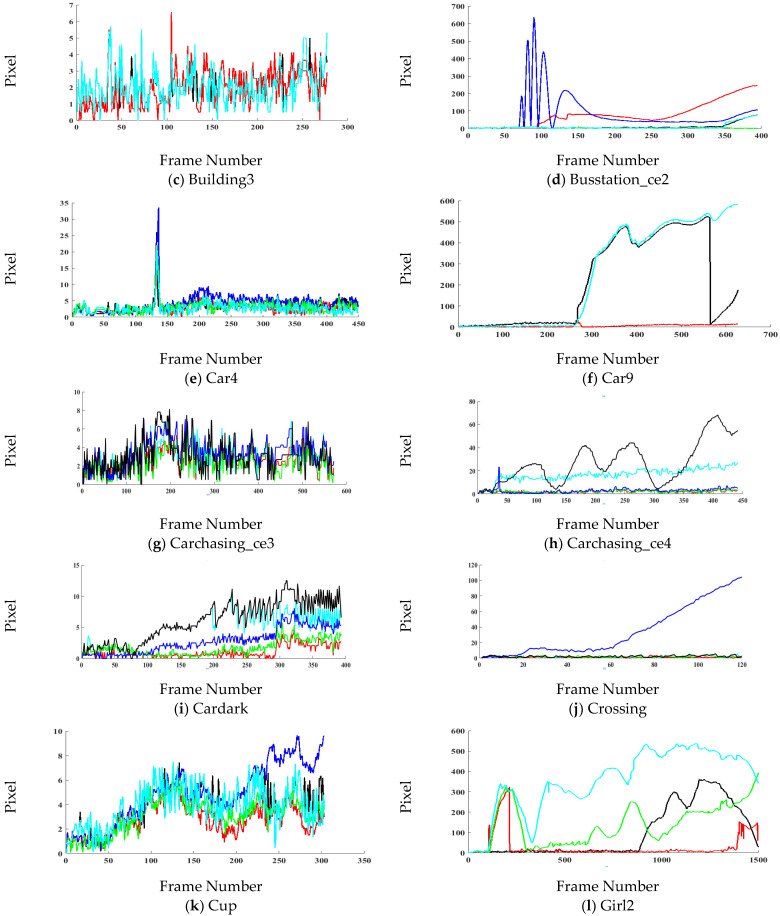

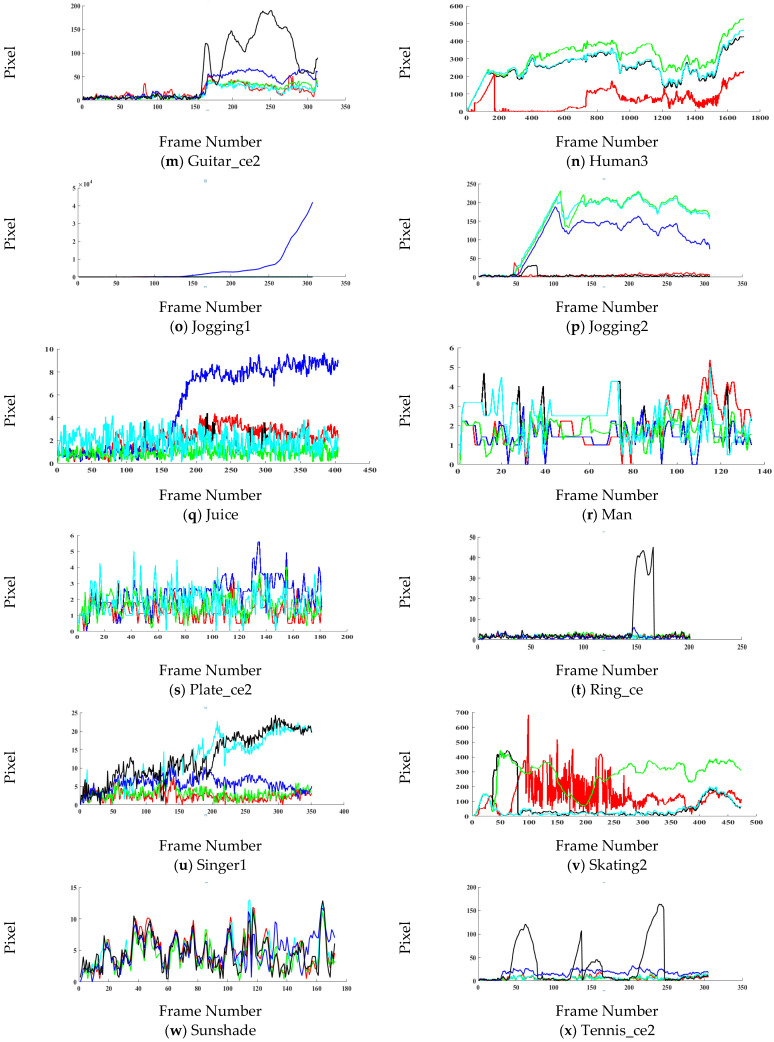

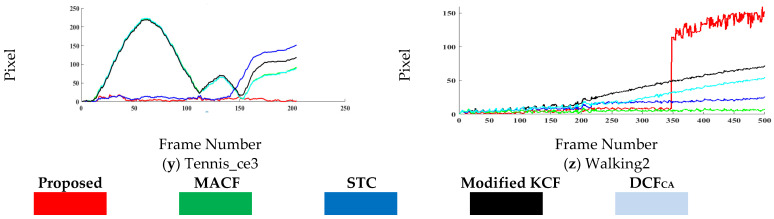
Center location error (in pixels) comparison on TC-128, OTB2013, OTB2015 and UAV123 datasets.

The location error plots are shown in Figure 4. In Table 3 average center location is calculated for each image sequence. It gives an idea about tracker performance, but it does not entirely incorporate all information necessary to review tracker performance. A possible scenario exists in object tracking when a tracker might drift for a few frames in a sequence resulting in a high error value. However, when the tracker recovers from drift and starts tracking the target accurately, the error will be low during those frames, but its average value will be high. Therefore, these plots are presented to review tracker performance on each frame. The proposed tracker performs consistently for sequences (Baby_ce, Car4, Car9, Cardark, Crossing, Carchasing_ce3, Carchasing_ce3, Cup, Guitar_ce2, Juice, Jogging2, Ring_ce and Tennis_ce3) over the entire duration. In sequences (Girl2, Human3, Skating2 and Walking2), the tracker gets drift between the frames but recovers after a few frames. For the majority of the frames in these sequences proposed tracker accurately tracks the target. However, when the tracker got drift, then the accumulative error is high for these sequences. In sequences (Bike3, Building3, Busstation_ce2, Jogging1, Man, Plate_ce2, Singer1, Sunshade, and Tennis_ce2) the proposed method has similar performance with compared trackers.

### 5.2. Qualitative Analysis

**Figure 5:**
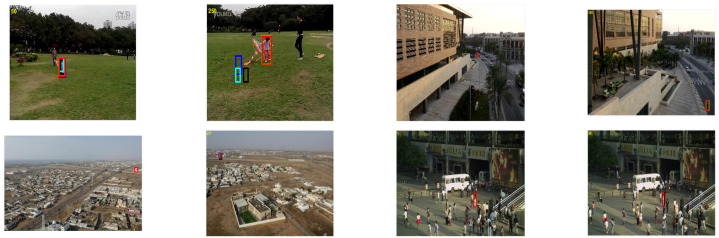

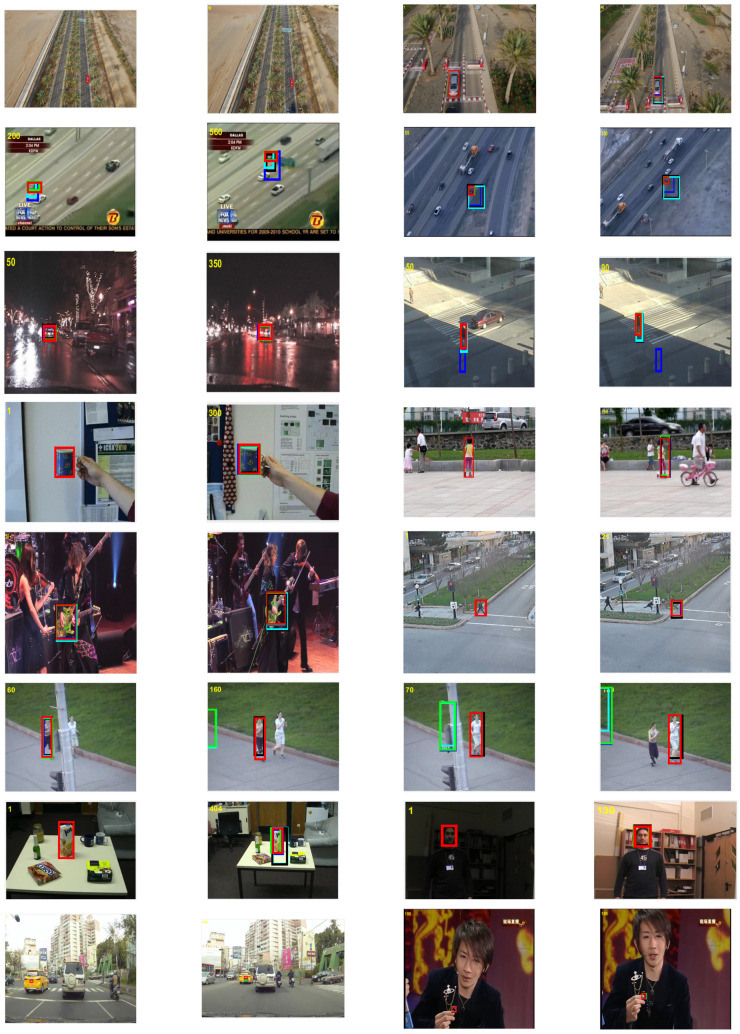

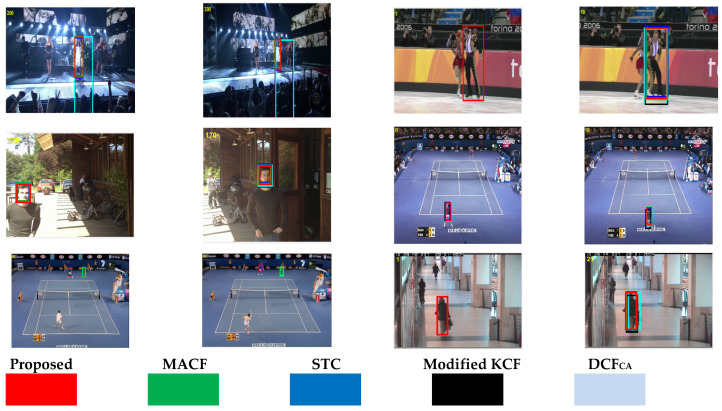
Qualitative comparison on TC-128, OTB2013, OTB2015, and UAV123 datasets.

Figure 5 depicts the proposed tracking qualitative results with four state-of-the-art trackers over 26 image sequences involving various challenges such as partial or full occlusions, scale variations, background clutter, etc. MACF contains a similar tracking component as our approach, i.e., scale correlation filter and Kalman filter. Even though MACF performs favorably well in sequences involving scale variations, it does not deal effectively with sequences involving occlusions (Girl2, Human3, Jogging1, Jogging2, and Skating2). STC uses intensity features and response of a single translation filter to estimate scale. This makes STC a comparatively fast tracker; however, there is no occlusion detection or handling mechanism due to which its tracking results are affected in sequences (Busstation_ce2, Girl2, Human3, Jogging1, and Jogging2). Moreover, due to only one translation filter, its tracking results are also affected (Car9, Crossing, and Tennis_ce3). DCF_CA_ contains correlation filtering combined with the context-aware formulation. However, it is not robust in occlusions, scale variations, and deformation challenges. Therefore, DCF_CA_ does not perform well in sequences (Car9, Carchasing_ce4, Girl2, Human3, Jogging1, Jogging2, Skating2, and Tennis_ce3). Modified KCF performs significantly well in sequences involving occlusions. However, it does not perform well in scale variation sequences (Baby_ce, Car9, Carchasing_ce4, Guitar_ce2, Ring_ce, Singer1, Tennis_ce2 and Tennis_ce3).

It can be seen that the proposed tracking method outperforms other trackers in these sequences. In sequences (Baby_ce, Car4, Carchasing_ce4, Crossing, Cup, Jogging1, Jogging2, Guitar_ce2, Plate_ce2, Ring_ce, Singer1, Tennis_ce2 and Tennis_ce3) the proposed method can accurately track the target for entire image sequences. In sequences (Bike3, Busstation_ce2, Girl2, Human3, Skating2, and Walking2) the tracker cannot accurately perform for the entire sequence. In sequences (Building3, Carchasing_ce3, Cardark, Juice, Man, and Sunshade), all trackers have similar performance.

## 6. Discussion

It can be seen from Figure 5 that the proposed tracking method outperforms other trackers in these sequences. We discuss several observations from performance analysis. This performance can be strengthened for three reasons. First, the scale correlation filter is incorporated in the STC framework making it deal effectively better than the STC scale. This scale filter learns target appearance on different scales, making it better to track targets accurately under scale variation scenarios. It can be seen in sequences (Baby_ce, Car4, Car9, Carchasing_ce3, Carchasing_ce4, Plate_ce2, and Ring_ce) that the proposed tracker deals better with scale variation of the target. Second, the incorporation of an extended Kalman filter makes it robust to handle occlusions. When the target undergoes partial or full occlusions, then EKF predicts the target state and updates the tracking model. It can be seen in sequences (Girl2, Jogging1, and Jogging2) that the proposed method can effectively handle the target’s occlusion. Third, the fusion of APCE based adaptive learning rate further elevates the tracking performance in illumination variations, motion blur, clutter background challenges. It can be seen in sequences (Building3, Cardark, Crossing, Cup, Guitar_ce2, Juice, Man, Singer1, Sunshade, Tennis_ce2 and Tennis_ce3) that the tracker can accurately follow the target. It is because the tracker’s appearance model can cope with changes in the environment by utilizing information in each frame.

Even though the proposed tracker performs significantly better than various trackers, there are few sequences (Bike3, Busstation_ce2, Human3, Skating2, and Walking2) in which the tracker does not track the target accurately. In Bike3 the tracker fails due to fast movement combined with scale variation. In Skating2 the tracker fails due to the deformation of the target. In (Busstation_ce2, Human3, and Walking2) the tracker fails due to occlusions, fast motion, and motion blur. The limitations can be addressed by working in few directions, such as developing a better occlusion detection and handling mechanism, extending the aspect ratio adaptability, and incorporating context-aware formulation.

## 7. Conclusions

This article gives insight into the robust tracking algorithm based on STC by incorporating scale correlation filter based on pyramid representation for adaptive scale estimation, extended Kalman filter for occlusion handling, and APCE criteria for the adaptive learning rate of the tracking model. Experimental results indicate that the proposed tracking algorithm performs better than the various state of the art qualitatively and quantitatively. The tracker achieved the desired performance, but the target may be lost in some cases like occlusions, motion blur, and fast motion. To address these limitations, our future work includes extending the current framework to context-aware and target adaptation formulation, development of occlusion judgment criteria, incorporation of more features to learn target appearance, and extending the aspect ratio adaptability.

## Figures and Tables

**Table 1:** List of notations/variables.

Symbol	Note
$H^{l}$	1-dimensional HOG feature extracted from the sample
$F_{t}^{l}$	Correlation filter at *t*th frame
$A_{t}^{l}$	The numerator of correlation filter at *t*th frame
$B_{t}$	The denominator of correlation filter at *t*th frame
$G_{t}$	2-dimensional Gaussian function
y	Response map of correlation filter
$x_{t}$	Predicted state at *t*th frame
$P_{t}$	Updated error covariance at *t*th frame

**Table 2:** Distance precision rate at the threshold of 20 pixels.

Sequence	Proposed	Modified KCF	Modified STC	STC	AFAM-PEC	MACF	DCF_CA_
Baby_ce	1	0.591	0.591	0.699	0.456	1	0.997
Bike3	0.206	0.166	0.296	0.275	0.124	0.275	0.262
Building3	1	1	1	1	1	1	1
Busstation_ce2	0.238	0.889	0.878	0.194	0.927	1	0.886
Car4	0.997	0.998	0.452	0.991	0.98	1	0.998
Car9	0.988	0.362	0.976	0.201	0.917	0.988	0.424
Carchasing_ce3	1	1	1	1	1	1	1
Carchasing_ce4	1	0.400	0.556	0.995	0.199	1	0.717
Cardark	1	1	1	1	1	1	1
Crossing	1	1	0.575	0.533	1	1	1
Cup	1	1	1	1	1	1	1
Girl2	0.830	0.591	0.372	0.262	0.940	0.097	0.071
Guitar_ce2	0.568	0.505	0.108	0.524	0.524	0.524	0.581
Human3	0.302	0.006	0.018	0.088	0.795	0.005	0.006
Jogging 1	0.879	0.993	0.996	0.228	0.973	0.231	0.231
Jogging 2	0.980	0.945	0.228	0.185	0.990	0.166	0.160
Juice	1	1	1	1	1	1	1
Man	1	1	1	1	1	1	1
Plate_ce2	1	1	1	1	1	1	1
Ring_ce	1	0.905	0.129	1	1	1	1
Singer1	1	0.815	1	1	1	1	0.843
Skating2	0.074	0.302	0.076	0.023	0	0.014	0.423
Sunshade	1	1	0.228	1	1	1	1
Tennis_ce2	1	0.656	0.101	0.652	1	1	1
Tennis_ce3	1	0.098	0.186	0.691	0.108	0.107	0.108
Walking2	0.694	0.408	0.934	0.442	0.722	1	0.564
**Mean Precision**	0.837	0.717	0.604	0.653	0.794	0.746	0.703

**Table 3:** Average center location error.

Sequence	Proposed	Modified KCF	Modified STC	STC	AFAM-PEC	MACF	DCF_CA_
Baby_ce	3.93	81.38	37.25	12.02	40.42	4.89	8.67
Bike3	131.33	123.70	86.37	81.97	75.20	83.58	87.73
Building3	2.02	1.96	1.79	1.50	1.97	3.76	2.02
Busstation_ce2	79.33	10.74	10.86	78.25	6.2	3.58	9.71
Car4	2.83	2.93	229.81	4.28	5.04	3.08	2.66
Car9	5.94	210.36	13.69	205.7	3.48	3.08	255.42
Carchasing_ce3	2.68	3.04	3.90	3.55	2.52	2.39	3.05
Carchasing_ce4	2.06	26.73	112.99	2.92	140	2.24	16.68
Cardark	1.03	6.04	3.21	2.83	3.35	1.67	5.11
Crossing	1.23	2.24	27.05	34.06	4.71	1.64	2.20
Cup	2.82	4.02	4.63	4.84	2.48	3.11	3.85
Girl2	30.79	98.93	101.77	200.5	9.32	137.62	356.78
Guitar_ce2	19.20	59.91	168.93	29.72	19.12	19.03	16.06
Human3	66.31	249.60	348.38	210.8	15.2	308.41	257.99
Jogging 1	18.34	3.72	8.40	5010	3.87	94.93	89.44
Jogging 2	5.46	4.74	43.04	104.02	5.09	148.98	148.33
Juice	2.16	1.96	4.63	5.08	2.42	0.91	1.92
Man	2.00	2.36	1.32	1.49	2.20	1.73	2.23
Plate_ce2	1.23	1.79	2.58	2.34	1.21	1.62	1.83
Ring_ce	1.56	5.21	69.55	1.30	1.71	1.80	1.68
Singer1	2.50	12.84	6.58	5.76	7.22	3.34	12.65
Skating2	142.18	78.67	69.79	106.33	200.4	277.60	46.90
Sunshade	4.91	4.54	68.68	4.99	4.57	4.20	4.84
Tennis_ce2	5.51	31.21	133.69	16.92	5.66	5.74	5.69
Tennis_ce3	5.78	97.29	79.58	40.73	91.1	90.95	90.72
Walking2	45.15	32.09	11.94	13.83	46.34	4.81	22.33
**Average Error**	22.63	44.54	63.48	237.91	26.95	46.72	56.02

**Table 4:** Frames per second (FPS).

Sequence	Proposed	Modified KCF	Modified STC	STC	AFAM-PEC	MACF	DCF_CA_
Baby_ce	14.35	101.87	34.68	93.20	27.98	59.2	100.77
Bike3	15.12	194.80	15.83	22.83	33.14	102.0	229.93
Building3	13.80	99.79	13.24	22.89	14.60	73.8	72.16
Busstation_ce2	9.14	56.57	10.07	38.67	30.08	32.4	47.50
Car4	7.13	97.91	11.15	23.16	13.01	58.6	76.33
Car9	2.40	15.77	4.59	29.23	10.44	14.1	19.39
Carchasing_ce3	26.47	165.00	54.62	139.47	36.88	78.3	125.51
Carchasing_ce4	7.33	4.77	11.51	60.62	11.53	32.6	16.40
Cardark	24.13	144.27	49.02	140.69	24.37	57.0	92.73
Crossing	19.01	72.64	47.80	112.10	39.69	34.5	38.44
Cup	10.23	33.67	22.24	76.77	15.98	47.6	16.57
Girl2	5.06	18.70	11.77	12.50	15.62	23.7	15.31
Guitar_ce2	0.92	3.70	1.83	12.10	5.16	4.68	10.60
Human3	10.49	14.54	19.48	131.02	21.18	38.6	46.84
Jogging 1	10.30	12.28	28.28	34.37	27.89	25.8	49.30
Jogging 2	7.32	23.31	16.81	38.23	22.51	30.0	14.97
Juice	9.96	39.36	19.99	99.70	20.24	38.6	38.78
Man	24.37	54.06	41.99	127.54	15.55	45.6	63.92
Plate_ce2	12.91	166.83	6.07	25.09	22.90	69.0	179.17
Ring_ce	19.18	126.04	33.45	67.68	15.99	65.1	125.46
Singer1	1.57	16.40	3.12	20.62	13.47	9.56	15.59
Skating2	1.13	10.94	2.69	58.00	15.28	8.05	16.18
Sunshade	10.81	41.02	30.97	75.39	25.98	40.1	36.15
Tennis_ce2	3.57	14.73	8.74	25.11	13.94	28.9	18.60
Tennis_ce3	6.70	19.81	12.44	28.72	22.99	58.2	48.47
Walking2	6.69	24.45	20.75	34.68	24.65	44.0	22.94

## Data Availability

Data is contained within the article.
